# Supplementary material for: From sequence to enzyme mechanism using multi-label machine learning
Source: BMC Bioinformatics. 2014 May 19;15:150. doi: 10.1186/1471-2105-15-150 (PMC4229970; doi:10.1186/1471-2105-15-150)
Supplement: Additional file 2 — Java code of ml2db. Additional file ml2db_code.tar.gz contains the Java source code to run the multi-label machine learning experiments and save the results to database. The code’s Javadoc is included. [file 1471-2105-15-150-S2.zip › additional file 2/ml2db/ml2db/doc/main/ml2db/weka/package-use.html]

Uses of Package main.ml2db.weka


JavaScript is disabled on your browser.


- Overview
- Package
- Class
- Use
- Tree
- Deprecated
- Index
- Help

- Prev
- Next

- Frames
- No Frames

- All Classes

# Uses of Package main.ml2db.weka

- Packages that use main.ml2db.weka

  | Package | Description |
  | --- | --- |
  |  |  |
  | --- | --- |
  | main.ml2db.mulan.attributesfiltered |  |
  | main.ml2db.mulan.mulanarff |  |
  | main.ml2db.mulan.mulanarff.database |  |
  | main.ml2db.weka |  |
  | test.ml2db.dataharness |  |
  | test.ml2db.weka |  |
- Classes in main.ml2db.weka used by main.ml2db.mulan.attributesfiltered

  Class and Description

  Arff

  Creates an ARFF file: checks file properties, loads data from database, fills
  and writes to file the ARFF attributes, classes and instances, generates
  auxiliary files if needed.

  ArffRecordsTable

  Class to store the name and column types for the database table which stores
  the ARFF records.
- Classes in main.ml2db.weka used by main.ml2db.mulan.mulanarff

  Class and Description

  Arff

  Creates an ARFF file: checks file properties, loads data from database, fills
  and writes to file the ARFF attributes, classes and instances, generates
  auxiliary files if needed.

  ArffProps

  A class containing the meta-data for a Weka ARFF file (or arff + xml files
  for Mulan ARFF) The data includes the path to an existing ARFF file, an
  optional data set name and description, and various statistics for the data
  (number of instances, class values, attributes)

  ArffPropsFromFile

  Contains the properties for a Weka ARFF file to be generated from database
  queries.

  AttributeFactory

  The attribute factory produces binary and numeric Weka attributes.

  DataSetDbLoader

  Loads data (instances, attribute values, class values) from database using
  the queries in the data Properties.

  DataSetGenerator

  Generates and fills the Weka instances.

  DataSetManager

  DataSet generates a collection of Weka instances (rows of the ARFF file),
  their attributes (column names) and their attribute values (cell values) The
  last attribute is the class attribute.

  ImportArff

  Class to import an existing Weka ARFF file to a record in the arff database table.

  InstancesFiller

  Generates and fills individual instances for a weka data set.

  InstAttValueMap

  Contains [instance identifier, attribute name, attribute value] tuples.
- Classes in main.ml2db.weka used by main.ml2db.mulan.mulanarff.database

  Class and Description

  ArffRecordsTable

  Class to store the name and column types for the database table which stores
  the ARFF records.
- Classes in main.ml2db.weka used by main.ml2db.weka

  Class and Description

  Arff

  Creates an ARFF file: checks file properties, loads data from database, fills
  and writes to file the ARFF attributes, classes and instances, generates
  auxiliary files if needed.

  ArffProps

  A class containing the meta-data for a Weka ARFF file (or arff + xml files
  for Mulan ARFF) The data includes the path to an existing ARFF file, an
  optional data set name and description, and various statistics for the data
  (number of instances, class values, attributes)

  ArffPropsFromFile

  Contains the properties for a Weka ARFF file to be generated from database
  queries.

  AttributeFactory

  The attribute factory produces binary and numeric Weka attributes.

  DataSetChecker

  Checks that all data (or file paths) needed to build the ARFF file are
  available

  DataSetDbLoader

  Loads data (instances, attribute values, class values) from database using
  the queries in the data Properties.

  DataSetGenerator

  Generates and fills the Weka instances.

  DataSetManager

  DataSet generates a collection of Weka instances (rows of the ARFF file),
  their attributes (column names) and their attribute values (cell values) The
  last attribute is the class attribute.

  DataSetWriter

  Class

  InstancesFiller

  Generates and fills individual instances for a weka data set.

  InstAttValueMap

  Contains [instance identifier, attribute name, attribute value] tuples.

  JaccardDistance

  This is an implementation of the Jaccard distance which calculates it on the
  fly.

  JaccardDistanceStored

  This is an implementation of the Jaccard distance which pre-calculates and
  stores the numbers needed.
- Classes in main.ml2db.weka used by test.ml2db.dataharness

  Class and Description

  ArffPropsFromFile

  Contains the properties for a Weka ARFF file to be generated from database
  queries.
- Classes in main.ml2db.weka used by test.ml2db.weka

  Class and Description

  Arff

  Creates an ARFF file: checks file properties, loads data from database, fills
  and writes to file the ARFF attributes, classes and instances, generates
  auxiliary files if needed.

  ArffRecordsTable

  Class to store the name and column types for the database table which stores
  the ARFF records.

  DataSetChecker

  Checks that all data (or file paths) needed to build the ARFF file are
  available

  DataSetDbLoader

  Loads data (instances, attribute values, class values) from database using
  the queries in the data Properties.

  DataSetGenerator

  Generates and fills the Weka instances.

  DataSetManager

  DataSet generates a collection of Weka instances (rows of the ARFF file),
  their attributes (column names) and their attribute values (cell values) The
  last attribute is the class attribute.

  DataSetWriter

  Class

  InstancesFiller

  Generates and fills individual instances for a weka data set.

  InstAttValueMap

  Contains [instance identifier, attribute name, attribute value] tuples.

- Overview
- Package
- Class
- Use
- Tree
- Deprecated
- Index
- Help

- Prev
- Next

- Frames
- No Frames

- All Classes
